# Comparing Adjuvanted H28 and Modified Vaccinia Virus Ankara Expressing H28 in a Mouse and a Non-Human Primate Tuberculosis Model

**DOI:** 10.1371/journal.pone.0072185

**Published:** 2013-08-19

**Authors:** Rolf Billeskov, Jan P. Christensen, Claus Aagaard, Peter Andersen, Jes Dietrich

**Affiliations:** 1 Department of Infectious Disease Immunology, Statens Serum Institut, Copenhagen, Denmark; 2 Institute of International Health, Immunology and Microbiology, University of Copenhagen, Denmark; University of Cape Town, South Africa

## Abstract

Here we report for the first time on the immunogenicity and protective efficacy of a vaccine strategy involving the adjuvanted fusion protein “H28” (consisting of Ag85B-TB10.4-Rv2660c) and Modified Vaccinia Virus Ankara expressing H28. We show that a heterologous prime-boost regimen involving priming with H28 in a Th1 adjuvant followed by boosting with H28 expressed by MVA (H28/MVA28) induced the highest percentage of IFN-γ expressing T cells, the highest production of IFN-γ per single cell and the highest induction of CD8 T cells compared to either of the vaccines given alone. In contrast, in mice vaccinated with adjuvanted recombinant H28 alone (H28/H28) we observed the highest production of IL-2 per single cell and the highest frequency of antigen specific TNF-α/IL-2 expressing CD4 T cells pre and post infection. Interestingly, TNF-α/IL-2 expressing central memory-like CD4 T cells showed a significant positive correlation with protection at week 6 post infection, whereas the opposite was observed for post infection CD4 T cells producing only IFN-γ. Moreover, as a BCG booster vaccine in a clinically relevant non-human primate TB model, the H28/H28 vaccine strategy induced a slightly more prominent reduction of clinical disease and pathology for up to one year post infection compared to H28/MVA28. Taken together, our data showed that the adjuvanted subunit and MVA strategies led to different T cell subset combinations pre and post infection and that TNF-α/IL-2 double producing but not IFN-γ single producing CD4 T cell subsets correlated with protection in the mouse TB model. Moreover, our data demonstrated that the H28 vaccine antigen was able to induce strong protection in both a mouse and a non-human primate TB model.

## Introduction

*Mycobacterium tuberculosis* (M.tb) is the causative agent of human tuberculosis (TB), a disease that is estimated to cause 1.5 million deaths a year [1]. Exposure to M.tb results in a quiescent latent infection in more than 90% of cases, and latent TB infection (LTBI) is estimated to be found in one third of the human population [2]. In about 10% of cases LTBI will reactivate resulting in active TB disease [1], [2]. The available vaccine, BCG, is an attenuated strain of *Mycobacterium bovis*. It efficiently protects children, but much less so adolescents or adults against TB in large well-documented field studies, and does not seem to prevent reactivation of LTBI by re-vaccination [3], [4], [5]. A new and improved vaccine is therefore needed.

It is well known that protection against M.tb infection is highly dependent on an efficient M.tb-specific Th1-response with CD4 T cells playing a critical role. In contrast, the role of CD8 T cells does not seem as crucial, although this is still under debate [6], [7], [8], [9], [10]. The pivotal role of IFN-γ and CD4 T-cells has been extensively documented in CD4^−/−^ and IFN-γ^−/−^ knockout (GKO) mice and mice either depleted of CD4 T cells or treated with anti-IFN-γ [9], [10], [11], [12]. Furthermore, people with gene-deficiencies impairing IFN-γ and IL-12 signaling pathways showed increased susceptibility to M.tb-infection [13]. Moreover, HIV-infection also increased the risk of reactivation from LTBI due to the depletion of CD4 T cells [14], [15]. As a result, most novel TB vaccine candidates have been developed to induce anti-M.tb Th1 immunity [16]. Recently, increasing effort has gone into identifying the most protective anti-TB Th1 subtypes. In several studies these subtypes were identified based on their cytokine expression profile [17], [18], [19]. Although far from fully resolved it has been shown that polyfunctional CD4 T cells expressing IFN-γ, TNF-α and IL-2 simultaneously are related to protection against intracellular bacteria like M.tb [18] and these polyfunctional T cells were also recently found to correlate with a favorable TB disease status in humans [20]. However, some studies did not find any correlation between the presence of certain polyfunctional T cell subtypes and protection against infection, leaving this important question not fully resolved [21], [22].

Regarding new vaccines against infection with M.tb, recombinant MVA expressing Ag85A (MVA85A) has been shown to boost and improve protection of BCG in mice, cattle and rhesus monkeys and to induce both CD4 and CD8 T cells [23], [24], [25]. In addition, clinical trials in both the UK and Africa have shown a capacity of MVA85A to boost T cell responses to BCG [26], [27], [28]. Thus, as a vector MVA85A has shown promise. However, it should be noted that not all studies show convincing results with MVA based vaccines and that the Ag85A antigen in MVA did not induce significant protection in a recent phase 2b trial in infants [23], [24], [29], [30], [31]. Another promising vaccine strategy is based on adjuvanted recombinant proteins. Thus, protein subunit vaccines in a Th1-adjuvant also have a long track record of protection against infection with M.tb in several species and some, such as Hybrid1 in IC31® [32], have entered clinical trials and shown an impressive memory response (reviewed in [16], [33]).

The objective of the study presented here was twofold: 1) to compare immunogenicity and efficacy of a recombinant protein in a Th1 adjuvant and/or protein expressed by Modified Vaccinia Virus Ankara (MVA) and 2) to identify immune correlates of protection with focus on protective T cell subtypes. These analyses were performed using a new TB vaccine antigen, HyVac28 (H28), composed of the TB antigens Ag85B, TB10.4 (previously tested as vaccine candidates in the fusion protein H4 [34]), and Rv2660c [35]. The vaccine was developed to induce late stage protection due to the antigen Rv2660c [36] and at the same time reserve the ESAT-6 antigen for diagnostics by replacing it with TB10.4. However, rather than performing a detailed comparison between H4 (Ag85B-TB10.4) and H28 (Ag85B-TB10.4-Rv2660c), the purpose of the present study was to compare immunogenicity and protection of H28 vaccine given as an adjuvanted subunit vaccine, an antigen expressed by MVA, or as a heterologous prime boost vaccine including both the MVA and the subunit approach.

**Figure 1:**
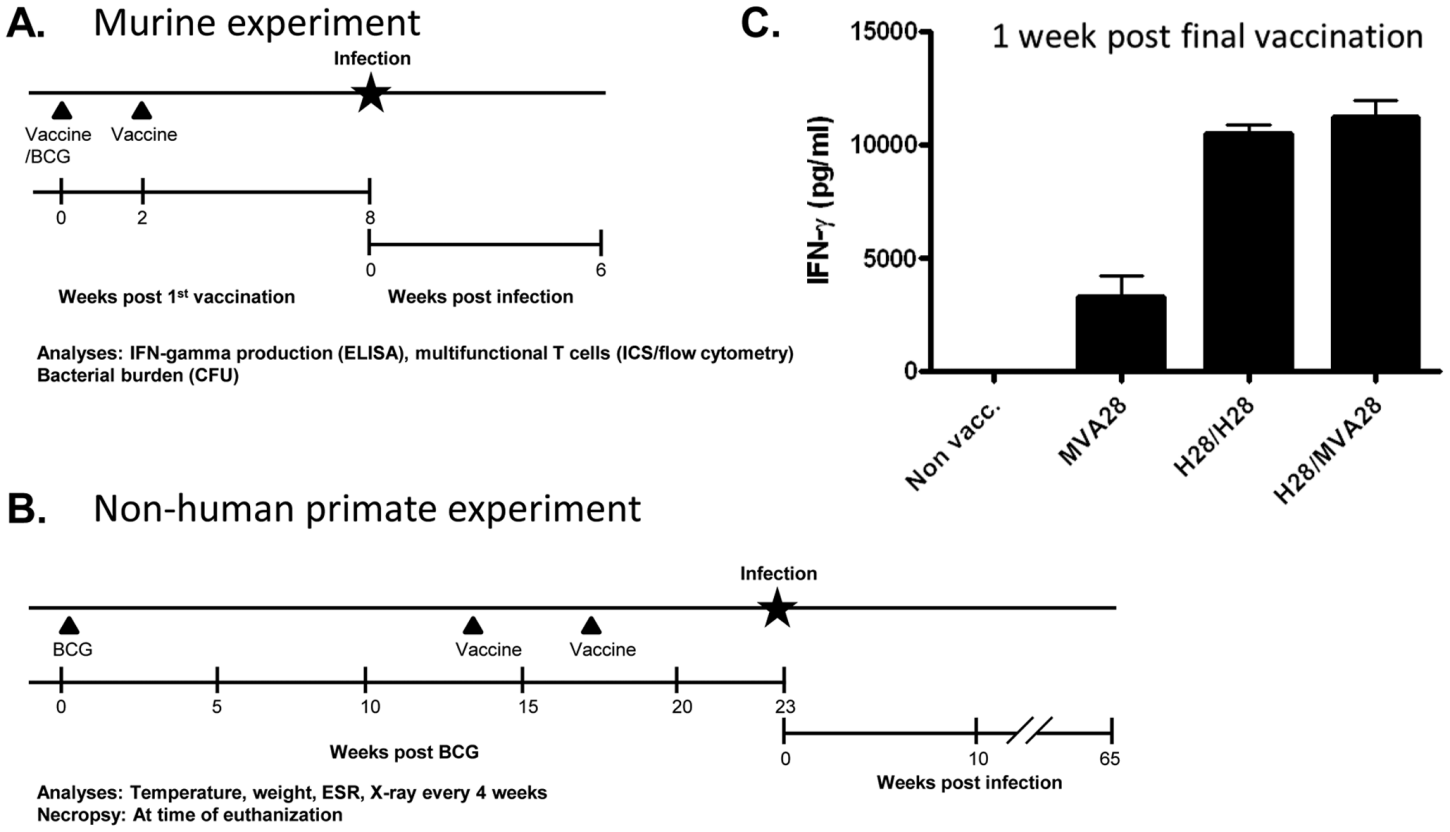
Using H28 expressed by Modified Vaccinia Virus or as a recombinant protein either alone or in a prime-boost regimen. A and B, a schematic overview of the murine and non-human primate experiments. C, mice were immunized subcutaneously two times with two weeks intervals as indicated. H28 was formulated in the adjuvant CAF01. IFN-γ production of PBMC’s stimulated with H28 was measured by ELISA one week after the final immunization. Bars represent means and standard deviations of triplicate cultures of pooled PBMCs from N = 9 animals.

The animal models chosen for this study were the mouse TB infection model as well as the cynomolgus macaque high dose TB model [37]. In the mouse model the vaccines were used as stand-alone vaccines without BCG priming as this allowed us to directly compare the vaccine induced Th1 subsets via multiparameter flow cytometry as well as their protective efficacy. In the non-human primate (NHP) model we chose the clinical parameters as readouts as these resemble human pathogenesis closely. Moreover, we used IC31® due to its excellent performance with another TB antigen in the NHP model [37] and finally, the vaccines were used as BCG-booster vaccines since we aimed to make the NHP study as clinically relevant as possible (see schematic overview in fig. 1A and B).

## Materials and Methods

### Methods Relating to the Mouse Experiments

#### Ethics statement

Experiments were conducted in accordance with the regulations set forward by the Danish Ministry of Justice and animal protection committees by Danish Animal Experiments Inspectorate Permit 2004-561-868 (of January 7, 2004) and in compliance with European Community Directive 86/609 and the U.S. Association for Laboratory Animal Care recommendations for the care and use of laboratory animals. The experiments were approved by the Statens Serum Institut IACUC headed by DVM Kristin E. Engelhart Illigen. The method of sacrifice was cervical dislocation.

#### Animal handling

Studies were performed with 6- to 8-wk-old female CB6F1 C57BL/6xBALB/c mice from Harlan, Scandinavia. Mice were housed in appropriate animal facilities at Statens Serum Institut, and infected animals were housed in cages contained within laminar flow safety enclosures (Scantainer, Scanbur, Denmark) in a separate biosafety level 3 facility at Statens Serum Institut.

#### Bacteria

M.tb Erdman were grown at 37°C on Middlebrook 7H11 (BD Pharmingen) agar or in suspension in Sauton medium (BD Pharmingen) enriched with 0.5% sodium pyruvate, 0.5% glucose, and 0.2% Tween 80. BCG Danish strain 1331 was grown at 37°C in Middlebrook 7H9 medium (BD Pharmingen). All bacteria were stored at −80°C in growth medium at ∼5×10^8^ CFU/ml. Bacteria were thawed, sonicated, washed, and diluted in PBS before the infection.

#### Antigens and vaccine vectors

The H28 gene fusion was constructed by amplifying the H4 gene product from the H4 expression vector [34] and the *rv2660c* gene from M.tb H37Rv chromosomal DNA followed by a second overlap PCR reaction. For all PCR reactions the iProf™ PCR system (BioRad) was used according to the manufacturer’s instruction. The H4 gene fusion was amplified using the primers 5′ - *GGGACAAGTTTGTACAAAAAAGCAGGCTTA*TCCC GGCCGGGGCTGCCG-3′ (Primer 1) and 5′- cgctatcacGCCGCCCCATTTGGCG - 3′ and the *rv2660c* gene using the primer set 5′-tggggcggcGTGATAGCGGGCGTCGACCA-3′ and 5′-*GGGGACCACTTTGT ACAAGAAAGCTGGGTC* CTAGTGAAACTGGTTCAATCCCAGTATCG-3′ (primer 2).

Recombination sites used for cloning are in italic, stop codon underlined and the sequence overlaps into *tb10.4* and *rv2660c* are in lower case. The resulting PCR products were purified and amplified for 10 rounds without primers followed by 25 rounds with primer 1 and 2 (0.3 uM each). The gel-purified fusion product (a*g85b-tb10.4-rv2660c*, *h28*) was cloned into the pDest17 expression by a 2-step recombination (Gateway™, Invitrogen). Recombinant H28 protein was expressed and purified from *E. coli* cultures as described for the H4 protein [34]. MVA expressing the H28 construct (MVA28) was produced by Bavarian Nordic using the MVA-BN **®** vector, based on the original replication deficient MVA developed by Anton Mayr and further passaged at Bavarian Nordic corresponding to passage number 598 [38]. *h28* from a pDEST17-plasmid was cloned into a recombination vector, where the *h28*-genes are controlled by the Pr7.5 early/late promoter, and selected by selection markers nptII and eGFP. The recombination plasmids were used for insertion of the *h28*-genes into MVA-BN® via homologous recombination. Recombinant MVA28 was propagated on primary chicken embryo fibroblasts (CEF) derived from Specific Pathogen Free (SPF) eggs.

#### Immunization

**Figure 2:**
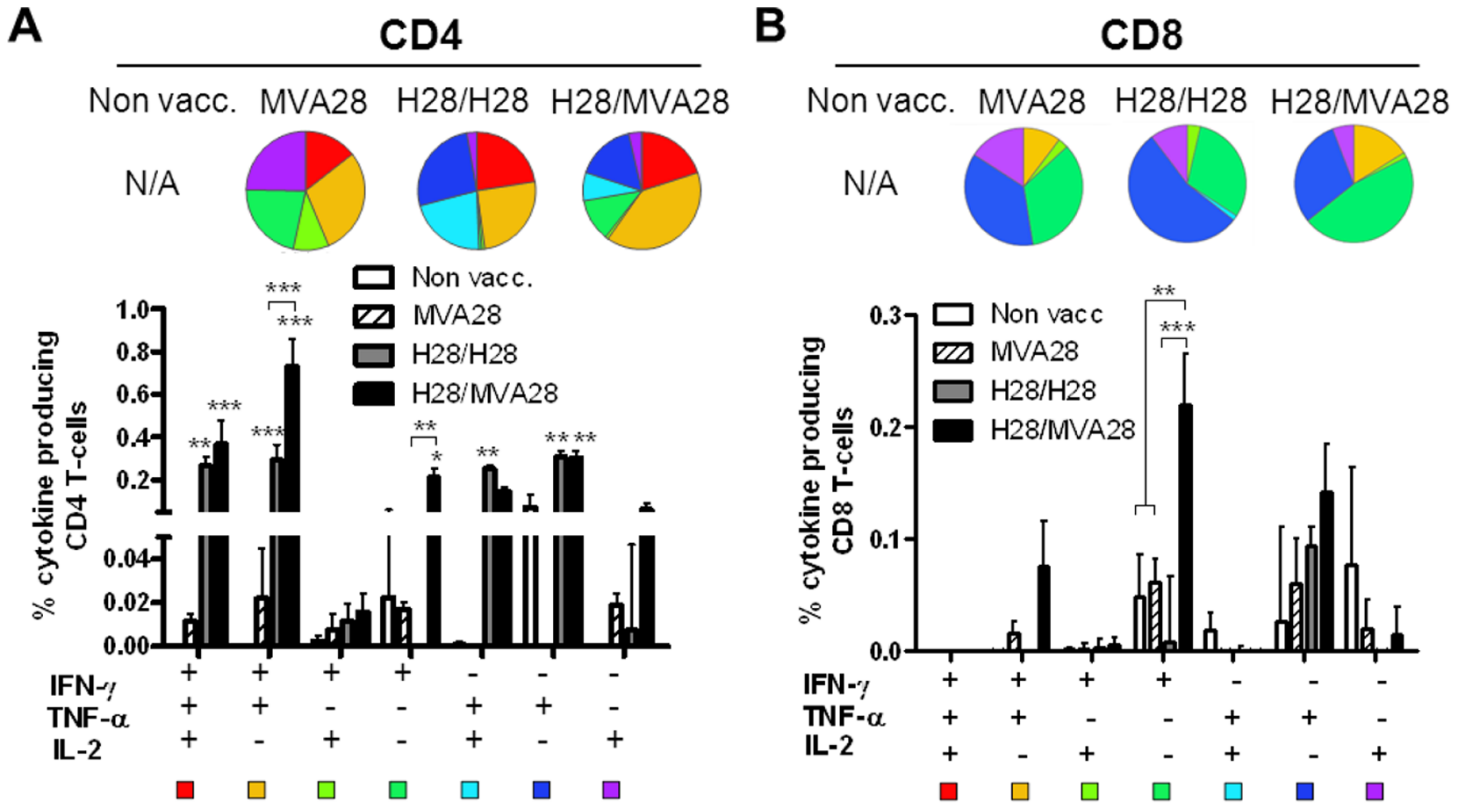
CD4 and CD8 T cell subsets post vaccination. One week after the second and final immunization splenocytes were analyzed for intracellular cytokine production by CD4 or CD8 T cells following stimulation with Ag85B (A) or TB10.4 (B). Ag85B was used to evaluate CD4 T cell responses since the highest CD4 T cell vaccine responses were directed against Ag85B shown in A, whereas the highest CD8 T cell responses were directed against TB10.4 shown in B. Mice receiving MVA28 only were given one immunization as this was the optimal regimen for this vector, not shown. The single MVA28 immunization was given simultaneously with the second vaccination. Bars represent percentages of CD4 or CD8 T cells from individual spleens from N = 4 mice per group producing any combination of IFN-γ, TNF-α or IL-2 as indicated below the graphs. Background levels obtained in media-stimulated samples have been deducted. Pie charts represent the relative distribution within the antigen specific CD4 or CD8 T cells pools of subsets producing different cytokine combinations as shown in the histograms.

Mice were immunized two times at two-week intervals subcutaneously (s.c.) at the base of the tail in weeks 0 and 2 of the experiments. The H28/H28 group received 5 µg recombinant H28 formulated in CAF01. Mice immunized with BCG received a single dose of 5×10^6^ CFU of BCG Danish 1331 s.c. in a volume of 0.2 ml at the base of the tail at the same time point as the first H28-immunization (week 0). Mice immunized with MVA28 only received one immunization of 5×10^7^ TCID_50_ in a volume of 0.1 ml given at the same time as the second H28 or MVA28 booster vaccination (week 2), except in the experiment shown in figure 1, where MVA28 was given twice at week 0 and 2. No difference was observed between 1, 2 or 3 MVA28-immunizations regarding immunity and protection as the MVA28 response was already fully developed after a single dose. The immune responses shown in figure 2 depict mice having received one MVA28 immunization one week prior to T cell analysis. For heterologous prime boost involving H28 protein and MVA28, mice were immunized 2 times as described above two weeks apart with one prime (H28) and one booster (MVA28).

#### Experimental infections

Mice were challenged by the aerosol route with ∼50–100 CFU of M.tb Erdman/mouse and infected six weeks after the last immunization as previously described [8 weeks after BCG; [39]]. Enumerations of bacterial colony forming units (CFU) in the lungs were determined by serial three-fold dilutions of whole-organ homogenates on 7H11 medium at six weeks after infection.

#### Lymphocyte cultures

Lymphocytes were cultured and purified as described previously [40]. Briefly, PBMCs were purified on a density gradient and splenocyte and lung lymphocyte cultures were obtained by passage through a cell strainer (BD Pharmingen). Antigens for stimulation were all used at 2 µg/ml. Supernatants from triplicate cultures (2×10^5^ cells per well) were harvested from cultures after 72 h of incubation for the investigation of IFN-γ.

#### Cytokine ELISA

A sandwich ELISA was used to determine the concentration of IFN-γ in culture supernatants as previously described [40].

#### Flow cytometric analysis

Intracellular cytokine staining (ICS) of T cells was done as described previously [41]. Briefly, 1–2×10^6^ cells were stimulated for 1 hour with 2 µg/ml antigen and subsequently 5 hours in the presence of 10 µg/ml brefeldin A (Sigma-Aldrich). Samples were then stained for surface markers (CD44, CD4 and CD8), permeabilized and subsequently stained for intracellular cytokine expression (IFN-γ, IL-2 and TNF-α) using the BD Cytofix/Cytoperm kit according to the manufacturers instructions. All samples were run on a BD FACSCanto flow cytometer, and results were obtained by analysis in Flowjo software (Treestar Inc.), and Spice and Pestle software [42].

#### Statistical methods

A significant difference in protective efficacy as well as differences in immune responses measured by intracellular flow cytometry of vaccines was evaluated using a one-way ANOVA. Differences between mean log_10_ CFU estimates as well as between individual T cell subsets were assessed by Newman-Keul’s post test for multiple comparisons. A value of *p<*0.05 was considered significant. Prism version 5 software (GraphPad) was used for analysis. Differences between polyfunctional T cell subsets among the groups were analyzed by two-way ANOVA with groups and T cell subsets as the variables. Correlations between mean log_10_ CFUs and responses from ICS flow cytometry were performed using Pearson’s product-moment correlation coefficient (r) and correlation test. The coefficient of determination (r^2^) shows the amount of variation shared by the two variables. Graphpad prism 5.0 software was used for analysis.

### Methods Relating to the Monkey Experiments

#### Ethics statement

All experimental manipulations and protocols were approved by the Leonard Wood Memorial (LWM) Center for Leprosy Research IACUC. All animals used in the study were housed in facilities accredited by the Philippine Association for Laboratory Animal Science (PALAS) in accordance with standards established in the Animal Welfare Act and the Guide for the Care and Use of Laboratory Animals. Daily personal care was given to animals by skillful animal caretakers, and the monkeys’ cages were enriched with toys to improve general animal welfare. Animals were sedated to minimize stress related with procedures. After infection, animals were followed closely. Humane endpoints were defined as weight loss that exceeded 20% body mass, or when animal welfare and behavior was significantly affected by the infection. Animals also had visual, auditory and olfactory contact with other animals, and received standard primate feed and fresh fruit on a daily basis and had access to water ad libitum. Animals were euthanized with an i.v. overdose of sodium pentobarbital.

#### Experimental vaccine

The vaccine antigen construct H28 (Ag85B-TB10.4-Rv2660c) was formulated with the adjuvant IC31®. Doses used were 50 µg of antigen protein in IC31® (total volume of 500 uL). The adjuvant IC31® consists of a mixture of the peptide KLK (NH2-KLKL5KLK-COOH) (1250 mmol/ml) and the oligodeoxynucleotide ODN1a (oligo-(dIdC)13) (50 nmol/ml) provided by Intercell AG.

#### Experimental animals

Adult, cynomolgus macaques (*Macaca fascicularis*) ranging in age from 1.6 to 3.7 years and weighing 2.2 to 3.2 kg were used. Before the commencement of the studies, the macaques underwent a rigorous battery of diagnostic and clinical procedures (e.g., physical examination, complete blood count (CBC) with differential, erythrocyte sedimentation rate (ESR), serum chemistry profile, thoracic radiography, and standard tuberculin skin testing (TST)).

#### Animal infection and clinical assessments after infection

Under sedation, cynomolgus macaques were inoculated intratracheally with 500 CFU per monkey in a 1.0 ml volume of virulent M.tb Erdman strain. The infection was allowed to proceed until the macaques reached disease states that spanned a spectrum from no apparent disease to advanced disease. Weight, temperature, and the Sediplast Westergren ESR were recorded monthly. Chest radiographs were taken monthly and then evaluated by a board-certified thoracic radiologist with extensive experience in pulmonary tuberculosis.

#### Immunization

Monkeys (N = 6) were primed with 0.1 mL of BCG Danish or injected with saline delivered intradermally. Thereafter the monkeys were vaccinated intramuscularly in the gluteal muscle 2 times with 50 µg of H28 formulated with the IC31® at week 14 and with H28 or MVA28 at week 17 post BCG. The group that received non-adjuvanted MVA28 received two intradermal immunizations simultaneously of 50 ul each to achieve a total volume of 100 ul with 0.5×10^8^ TCID_50_). At week 23 the animals were challenged with M.tb and followed for 64 weeks.

#### Necropsy procedures and pathology scores

Animals were anesthetized with a cocktail of Zoletil 50+ Atrosite+Zylazil-100 (5∶1∶1) or ketamine given i.m at 0.1 ml/kg body weight and euthanized with an i.v. over-dose of sodium pentobarbital. Necropsy procedures were as follows: The thoracic cavity was entered, and the gross extent of mycobacterial infection was recorded. Each lung was examined for superficial lesions, and the gross dissemination of mycobacterial infection (e.g. number of visible granulomas) and other pathological findings was recorded for right and left lung separately, spleen, liver, kidney, thoracic lymph node and other sites (pericardium, stomach, bone). Scoring of lungs and other organs were recorded separately taking into account both the number and size of the lesions. i) *Number of lesions*. Score 0: no visible granulomas; score 1: 1–3 granulomas; score 2: 4–10 granulomas; score 3: >10 granulomas; score 4: miliary pattern with numerous lesions. ii) *Lesion size*: Score 1: 1–2 mm; score 2, 3–4 mm: score 3, >4 mm. Each organ (with pathological changes) could obtain a minimal score of 2, e.g. 1–3 small lesions (1–2 mm), and a maximum score of 10, e.g. miliary TB with several coalescent large granulomas. All individual pathology scores are recorded in Table S2.

#### Statistical analysis

For X-ray and pathology the animal groups were compared with a 1-way Anova, Kruskal-Wallis/Dunn’s Multiple Comparison Test. Survival analysis: Analysis of Kaplan Meier curves was performed with Log-rank (Mantel-Cox) Test. Since survival of 4 groups were compared to each other in the NHP study this resulted in a total of 6 comparisons, and we therefore used the Bonferroni correction for multiple comparisons, because the probability of a type 1 error (false-positive) was 0.05 for each comparison [43]. Thus, the Bonferroni corrected significance level (α-corrected) for each individual comparison equaled the significance level α ( = 0.05) divided by the number of comparisons **n** ( = 6), i.e. 0.00833. If a P value was less than this Bonferroni-corrected threshold (0.0083), then the comparison was considered statistically significant. All tests were done using Prism Software (GraphPad Software, La Jolla, CA, USA).

## Results

### Using H28 Expressed by Modified Vaccinia Virus or Adjuvanted Recombinant Protein in Homologous and Heterologous Prime-boost Vaccine Strategies

The first objective was to determine the vaccine strategy that induced the strongest Th1 response since a Th1 response is crucial in preventing infection with M.tb. We therefore tested different vaccine strategies involving H28 protein alone in a Th1-inducing adjuvant CAF01 and/or MVA expressing the H28 vaccine antigen. We chose the Th1 adjuvant CAF01 due to its efficient priming of polyfunctional CD4 T cells in mice [17].

Mice were immunized with H28/CAF01 (H28/H28), MVA expressing the H28 fusion protein (MVA28), or a heterologous prime boost combination of these two vaccines (H28/MVA28) (fig. 1A). The mice received two immunizations spaced by two weeks. The overall level of the vaccine response was measured one week after the second vaccination by IFN-γ ELISA on supernatants from PBMCs stimulated with the H28 fusion protein *in vitro* (fig. 1). The results showed that all three immunized groups induced a vaccine specific response, and that the highest response was observed in the heterologous prime-boost group and in mice vaccinated with the adjuvanted H28-protein alone. MVA28 alone gave the lowest response after H28 stimulation *in vitro* (MVA28: 3303±1555 pg/ml, H28/H28: 10507±625 pg/ml, H28/MVA28: 11231±1276 pg/ml; fig. 1C). Finally, priming with MVA28 and boosting with H28 induced significantly lower IFNγ-production compared to the H28/MVA28 regimen (MVA28/H28: 6621±1836 pg/ml IFNγ, data not shown).

### The T cell Phenotypes Induced by the H28 and MVA28 Vaccine Strategies

We next evaluated the level of polyfunctional CD4 T cell subsets induced by the different vaccine regimens in order to later examine the correlation of these subsets with protection against infection with M.tb. Mice were immunized as described above. Following the second vaccination, splenocytes were stimulated *in vitro* with the antigens Ag85B and TB10.4 and polyfunctional T cells were evaluated by intracellular cytokine staining and flow cytometry. The results showed that following Ag85B-stimulation, the group immunized twice with adjuvanted H28 protein induced the highest frequency of IL-2^+^TNF-α^+^ CD4 T cells with a proposed central memory-like [18] phenotype (0.25% of all CD4 T cells as shown in the histograms versus 0.14% in the H28/MVA28 group respectively; fig. 2A). Although this difference was not statistically significant, the H28/H28 group induced a significantly higher proportion of IL-2^+^TNF-α^+^ cells within the Ag85B specific CD4 T cell pool compared to the H28/MVA28 group (H28/H28: 21.9% of Ag85B specific CD4 T cells, H28/MVA28: 7.8% as shown in the pie charts, fig. 2A, p<0.05). In contrast to H28/H28, MVA28 and H28/MVA28 vaccines predominantly induced IFN-γ producing T cells, either IFN-γ single positive T cells (most probably terminally differentiated effector cells [18]), or polyfunctional T-cells expressing IFN-γ/TNF-α/IL-2 or IFN-γ/TNF-α (fig. 2A). Similar results were observed after TB10.4 stimulation, although at lower total frequencies (fig. S1). As expected [36] responses against Rv2660c constituted the lowest responses (data not shown) and consequently only Ag85B and TB10.4 were used throughout the paper to evaluate the different vaccination strategies.

Boosting H28 with MVA28 in the H28/MVA28 group significantly increased the amount of IFN-γ^+^, IFN-γ^+^TNF-α^+^ effector T cells, but not IFN-γ^+^TNF-α^+^IL-2^+^ or TNF-α^+^IL-2^+^ CD4 T cells (fig. 2A). Interestingly, CD4 T cells induced by H28/MVA28 immunization produced the highest amounts of IFN-γ per single cell compared to the other groups (P<0.05; measured by cytokine MFI), whereas CD4 T cells from the H28/H28 group induced significantly higher IL-2 production per single cell than CD4 T cells from the MVA28 and H28/MVA28 groups (p<0.05; fig. S2).

Concerning CD8 T cells, mice immunized with H28/MVA28 induced the highest CD8 response predominantly composed of CD8 T cells specific for TB10.4 (co-expressing IFN-γ and TNF-α; fig. 2B) and to a lesser extent Ag85B (fig. S3).

Thus, H28/MVA28-immunized mice showed the highest overall percentage of IFN-γ producing T cell subsets as well as the highest expression of IFN-γ per single cell, whereas H28/H28 mice contained the highest proportion of TNF-α^+^IL-2^+^ central memory-like CD4 T cells, and the highest expression of IL-2 per single cell (within the pool of antigen specific CD4 T cells). MVA28 given alone induced the lowest responses, and the cells induced by MVA28 primarily belonged to IFN-γ expressing phenotypes. These results were reproduced in 3 independent experiments (not shown).

### Protection against M.tb Infection in a Mouse TB Infection Model

**Figure 3:**
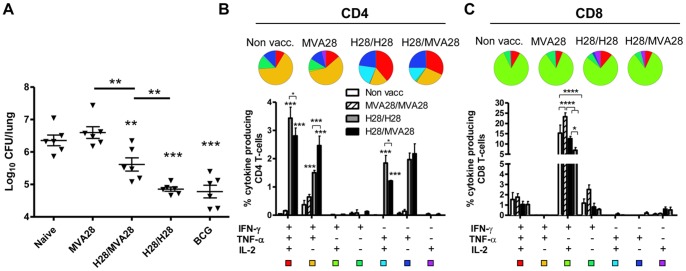
Protection in a mouse TB infection model and correlation with post-exposure T cell subsets. Mice were immunized with recombinant vaccines as described in figure 2. The BCG group was immunized once at week 0 (the same time as the first immunization with H28). A, six weeks after the final vaccinations (8 weeks after BCG) mice were subjected to aerosol infection with M.tb, and the bacterial burden was determined six weeks after infection. Data points represent individual mice and show the mean and SEM of estimated log_10_ values from N = 6 mice per group *p<0.05, **p<0.01, ***p<0.001 as indicated in the graph using one-way ANOVA and Newman-Keul’s post-test for multiple comparisons. Significance levels against the non-vaccinated group are indicated with asterisks above groups, other significant differences between vaccine groups are indicated by a line. B and C, intracellular cytokine analysis was performed by flow cytometry on lung cells six weeks after infection at the same time as mice were assessed for protection. Cells were stimulated with Ag85B for CD4 (B) and TB10.4 for CD8 (C) T cell analysis. Bars represent percentages of CD4 (B) or CD8 T cells (C) from individual lungs from 3 mice per group producing any combination of IFN-γ, TNF-α or IL-2 as indicated below the graphs. Background levels obtained in media-stimulated samples have been deducted. Pie charts represent the relative distribution of CD4 or CD8 T cells subsets producing different cytokine combinations as shown in the histograms.

We next studied the protective potential of the different vaccine strategies, adjuvanted H28, H28 expressed by MVA or the prime-boost regimen, in a mouse TB infection model. Six weeks after the last vaccination animals were infected by the aerosol route with virulent M.tb Erdman. Bacterial numbers were measured in the lungs of the animals at week six post infection. We found that the protection induced by the different vaccination strategies was as follows: H28/H28 ( = BCG)>H28/MVA28>MVA28 (fig. 3). Thus, H28/H28 protected significantly better than the H28/MVA28 group (1.50±0.07 log_10_ CFU protection ± SEM in H28/H28 mice versus 0.74±0.20 in H28/MVA28 mice, p = 0.01), and MVA28 induced no protection, or only a minor level of protection when 4 experiments were pooled (0.28±0.12 log_10_ CFU protection ± SEM, p<0.05; fig. 3 and S4). There was no difference in protection between BCG and H28/H28.

### Vaccine Induced T cell Subsets Post Infection

The recruitment of CD4 T cell subsets to the lungs after infection was also examined by flow cytometry in order to relate specific T cell subsets to protection. Six weeks post infection the H28/H28 group showed significantly higher percentages of IFN-γ^+^TNF-α^+^IL-2^+^ and TNF-α^+^IL-2^+^ Ag85B specific CD4 T cells (fig. 3B) compared to all the remaining groups. In contrast, in the H28/MVA28 group we observed the highest percentage of IFN-γ^+^TNF-α^+^ (p<0.001 compared to H28/H28 mice) and IFN-γ^+^ effector CD4 T cells specific for Ag85B. The same overall picture was seen for CD4 responses against TB10.4 (fig. S3). Importantly, the larger amount of TNF-α^+^IL-2^+^ and IFN-γ^+^TNF-α^+^IL-2^+^ specific CD4 T cells in turn correlated with lower bacterial numbers in the lung of H28/H28 vaccinated mice compared to the H28/MVA28 (p<0.01 comparing log_10_ CFU values), MVA28 vaccinated (p<0.001), or non-vaccinated (p<0.001) mice. When the CD8 T cell responses against TB10.4 were compared, we observed no major differences in the induced subsets by the different vaccines as illustrated by the very similar pie charts in figure 3C. The level of the CD8 response showed a positive correlation with the bacterial levels in agreement with previous studies suggesting that CD8 T cell responses post-exposure can be used as a marker for the severity of the infection [40] (fig. 3). Only low CD8 T cell responses were observed against Ag85B (fig. S3).

### Correlating T cell Subsets Pre- and Post Infection with Bacterial Levels

**Figure 4:**
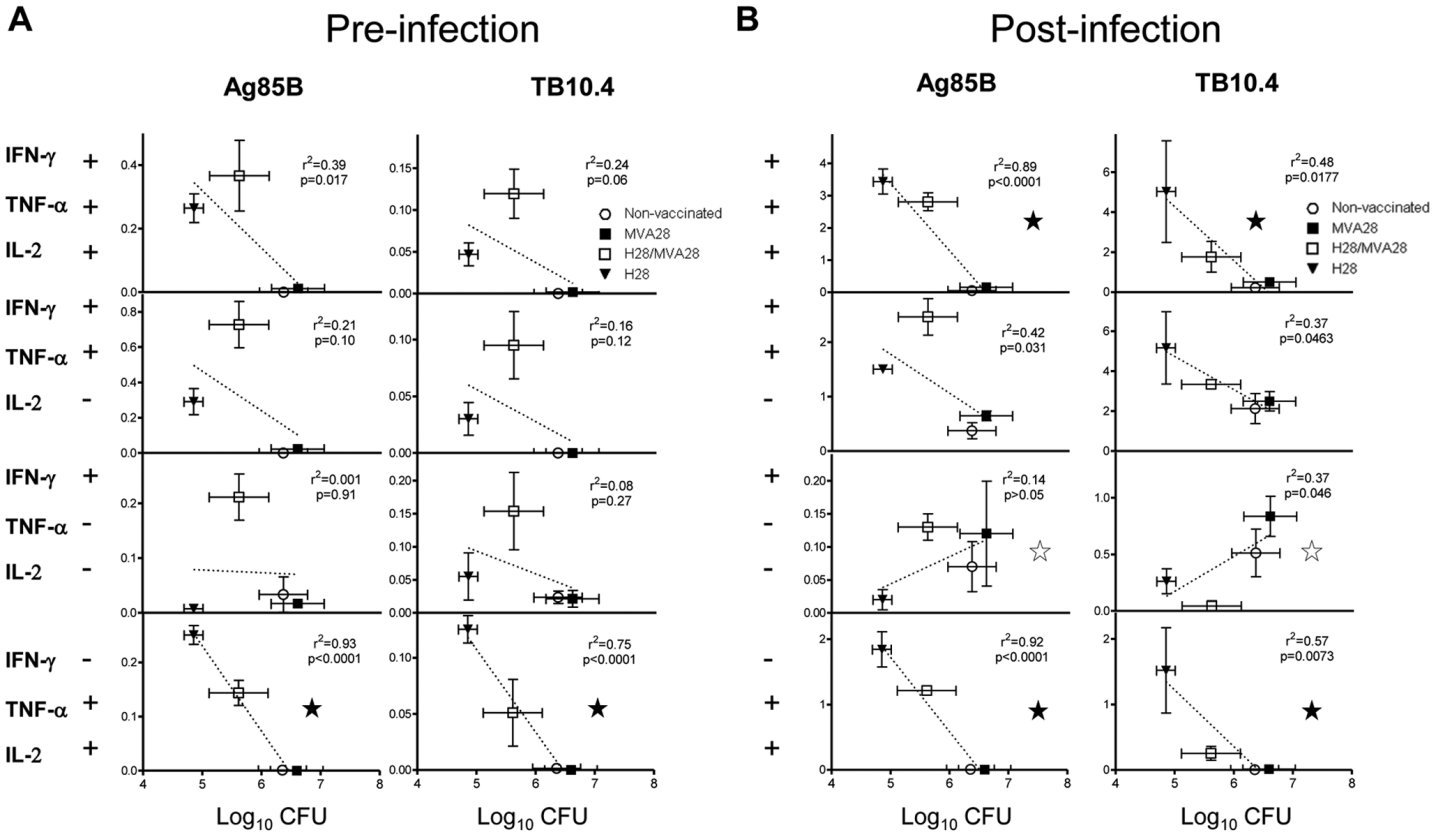
Correlation between T cell subsets induced pre and post infection and CFU. A, The splenic CD4 T cell responses obtained using ICS and flow cytometry one week after immunizations shown in figure 2 and S2 (Ag85B- and TB10.4-stimulation) were correlated to the corresponding mean log_10_ CFU value obtained in the lungs six weeks after infection. Points represent mean percentage +/− SEM (vertical) of CD4 T cells producing different combinations of IFN-γ, TNFα, IL-2 in response to stimulation with indicated antigens from 4 spleens per group plotted on the y-axis and mean +/− SEM (horizontal) log_10_ CFU values of individual mice on the x-axis. Each point represents one group as indicated in the graph. B, the pulmonary CD4 T cell responses obtained using intracellular cytokine staining (ICS) and flow cytometry six weeks after infection shown in figure 3 and S3 (Ag85B- and TB10.4-stimulation) were correlated to the corresponding mean log_10_ CFU values within each group. Points represent individual mean +/− SEM values as described in A. *p<0.05, **p<0.01, using Pearson’s product-moment correlation coefficient (r) and correlation test. T cell subsets that showed negative correlation with CFU are marked with a black star, whereas those that showed positive correlation are marked with a white star.

As the difference in CD4 T cell subset induction by the different vaccines correlated well with the level of protection, we decided to perform a correlation analysis by linear regression between IFN-γ^+^TNF-α^+^IL-2^+^, IFN-γ^+^TNF-α^+^, IFN-γ^+^, and TNF-α^+^IL-2^+^ antigen specific CD4 T cells (as these were the most prominently induced subsets). Both systemic responses pre infection in the spleen as well as pulmonary responses post infection in the lung were correlated to the bacterial log_10_ CFU levels obtained in the lungs six weeks post infection (fig. 4). The analysis showed that the pre infection CD4 T cell subset that showed the highest correlation with protection was the TNF-α^+^IL-2^+^ central memory-like [18] CD4 subset specific for both Ag85B and TB10.4 (fig. 4A). Thus, there was a strong negative correlation between the amount of TNF-α^+^IL-2^+^ CD4 T cells induced by the vaccines and the level of bacteria in the lungs 6 weeks after infection (R^2^ = 0.93 and 0.75, and p<0.0001 for Ag85B and TB10.4, respectively). Six weeks after infection there was still a strong negative correlation between TNF-α^+^IL-2^+^ CD4 T cells and the level of bacteria (R^2^ = 0.92 and 0.57, and p<0.0001 and p = 0.0073 for Ag85B and TB10.4, respectively; fig. 4B). Interestingly, at six weeks post infection in the lungs, there was also a negative correlation between IFN-γ^+^TNF-α^+^IL-2^+^ and IFN-γ^+^TNF-α^+^ CD4 T cells and bacteria levels. Moreover, IFN-γ^+^ single producers, most likely terminally differentiated T cells, correlated positively with CFU six weeks post infection. Regarding CD8 T cell responses, we observed a significant positive correlation between post infection TB10.4 CD8 T cell responses and log_10_ CFUs as described above (R^2^ = 0.64, p = 0.003, fig. S5). However, the level of CD8 T cell response pre infection did not correlate with CFUs implying that CD8 T cells were not important for protection.

### Evaluating H28 and MVA28 Vaccine Strategies in a Non-human Primate TB Model

We next examined the two best H28-based vaccination strategies observed in the murine studies, adjuvanted H28 (H28/H28) and the heterologous H28/MVA28 prime-boost regimen, in a high dose (500 CFU) tuberculosis NHP model using cynomolgus macaques (see figure 1B for an overview of the experiment). Since a novel recombinant TB vaccine most probably will be used as a BCG booster vaccine, we decided to test the vaccines as BCG booster vaccines in an NHP-model that recapitulates the human pathogenesis of TB very closely [44]. As adjuvant, IC31® was used as it was recently demonstrated to be protective with another subunit TB vaccine in the same monkey model [37]. Moreover, IC31® is presently evaluated in clinical trials with other TB vaccines [45]. As control groups, the monkeys were vaccinated with BCG or left untreated. These control groups of the current NHP-study have previously been published together with other vaccine groups [37].

### X-ray Changes Post Infection

**Figure 5:**
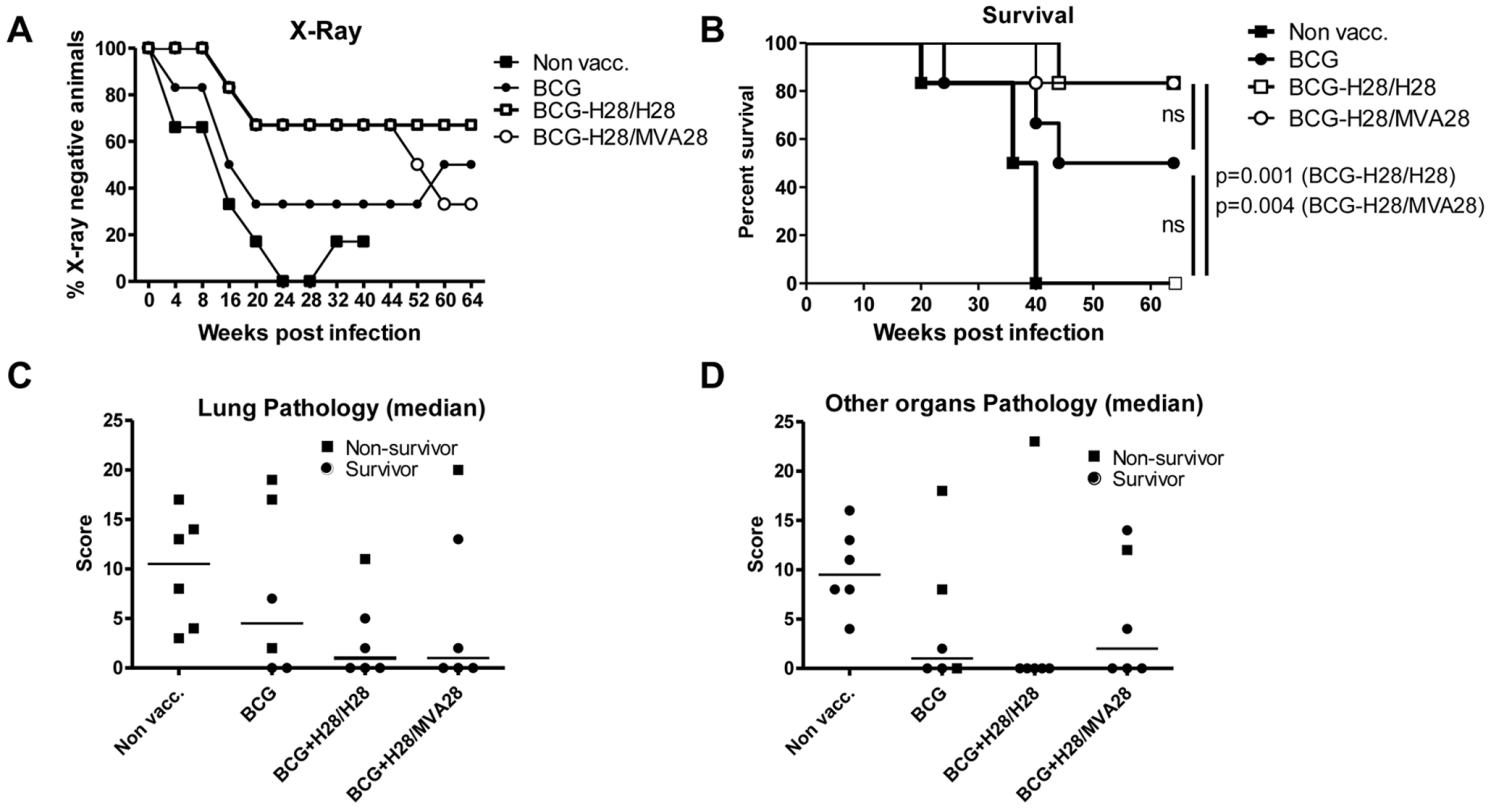
Clinical outcome and survival in a non-human primate TB model. A, the percentage of X-ray negative animals post-challenge. Non-vaccinated (black squares), BCG vaccinated (black circles), BCG vaccinated H28/H28 boosted (white squares), and BCG vaccinated H28/MVA28 boosted (white circles). The adjuvant used with H28 was IC31®. Individual X-ray findings are shown in Table S1. If an animal was positive and then had to be euthanized it remained positive in our summation for the curves depicted. B, animals were examined clinically and euthanized according to pre-established humane endpoints to generate the survival curve. C and D, pathology scores of lungs (C) and other organs (D). Medians are depicted by horizontal lines. Individual organ scores are given in Table S2. The figure shows the scores for both euthanized (filled boxes) and surviving (filled circles) animals.

The monkeys (N = 6 per group) were infected with M.tb (500 CFU) followed from baseline up to week 64 post infection (fig. 5). BCG-primed and non-vaccinated animals showed the first radiological symptoms of TB (four weeks post M.tb challenge), and throughout the experiment these groups had a very similar development of X-ray changes affecting 66–100% of monkeys from week 20 and onwards (fig. 5A). The main radiological diagnosis in these groups was bilateral progressive bronchopneumonia often with multiple nodules (6 out of 6 in non-vaccinated animals, and 4 out of 6 in BCG vaccinated animals) or even (in two of the animals) consolidation/atelectasis of major parts of the lung. In the BCG group boosted twice with IC31® adjuvanted H28 (BCG-H28/H28), only two animals had any detectable X-ray abnormalities (bilateral bronchopneumonia at week 20; fig. 5A and Table S1). In the BCG-H28/MVA28 group we also observed reduced radiological changes compared to the non-vaccinated and BCG groups, except for the very late stages of the infection (week 52 to 64 post infection). Thus, four animals developed unilateral bronchopneumonia (and one animal, 6496 D, as late as week 64 post infection). Animals in the non-vaccinated group had a progressive development of disease and all had to be euthanized before week 40 (mean survival time 37.3 weeks, fig. 5).

### Survival

In the BCG group, animals displayed a less aggressive course of infection than the non-vaccinated animals with 3/6 (50%) monkeys surviving throughout the 64 weeks observation period (mean survival time 49.3 weeks). Based on Kaplan-Meyer analysis this difference was not significant compared to the control group (0/6 survivors, P = 0.04, Log-rank Mantel-Cox using the Bonferroni-corrected significance threshold which, due to 6 multiple comparisons, equals 0.05/6 = 0.0083). In the BCG-H28/H28 group, 5/6 (83%) monkeys survived until the termination of the experiment (mean survival time 60 weeks), and this was significantly different from the non-vaccinated animals (P = 0.001; fig. 5B) but not from the BCG group. Likewise the mean survival time for the BCG-H28/MVA28 group (5/6 survivors) was increased (59.3 weeks) compared to the non-vaccinated group (P = 0.004) and the BCG group (not significant).

Thus, boosting BCG with H28/H28 or H28/MVA28 reduced X-ray changes and increased survival. The reduction in X-ray changes was slightly more pronounced in the BCG-H28/H28 group.

### Pathology Scores at Necropsy

At necropsy, all control animals from the non-vaccinated group showed extensive lung pathology, characterized by the presence of multiple granulomas in lungs and in a higher pathology score for the other organs (i.e. heart, spleen, liver and kidney). The median pathology score in the non-vaccinated group was 10.5 in both the lung and in other organs but the variation was significant with some animals having substantial pathological changes (fig. 5 C, D and Table S2). The BCG-only group did not show statistically significant differences in pathology compared to the control group using this scoring system. In contrast, the 5 surviving animals in the BCG-H28/H28 group all showed a pathology score of less than 5 in the lungs (3 animals scored 0), and remarkably none of the surviving animals showed any pathology outside the lung (non-vaccinated versus H28 boosted; p<0.05; fig. 5D). The BCG-H28/MVA28 group also showed reduced pathology scores compared to the BCG group (not statistically significant). However, the scores were slightly elevated (50% of the animals scored 0 both in the lung and in other organs) compared to the BCG-H28/H28 group (50% scored 0 in the lung and 83% scored 0 in other organs).

Taken together, boosting BCG with H28/MVA28 or H28/H28 reduced pathology in the lungs and reduced (in the BCG-H28/MVA28 group), or even completely prevented (in the BCG-H28/H28 group) dissemination to other organs in surviving animals.

## Discussion

The goal of the present study was to identify the best vaccine strategy, based on two well-known technologies – adjuvanted subunit vaccine and recombinant MVA expressing the vaccine antigens, in the mouse and NHP TB models. Moreover, immune analysis in the mouse model was performed to identify immune correlates of protection with focus on T cell subsets. H28 was used as the model antigen to achieve these goals. As Rv2660c is only poorly immunogenic in the CB6F1 mouse strain [36] we used the two other antigens, Ag85B and TB10.4, to perform the analysis of the immune response.

In the mouse model we found an interesting difference between the H28/H28 and H28/MVA28 vaccine strategies in terms of the induced T cell subsets. The heterologous prime-boost vaccination regimen H28/MVA28 induced the highest level of IFN-γ producing effector CD4 T cell subsets (fig. 1 and 2) and the highest expression of IFN-γ per single cell (fig. S2). In contrast, following vaccination with only H28 (in CAF01) we observed the highest proportion of the central memory-like TNF-α^+^IL-2^+^ CD4 T cell subset, and the highest expression of IL-2 per single cell (within the pool of Ag85B specific CD4 T cells, fig. 2, S1 and S2). This is in agreement with results obtained with an adenoviral vector expressing the HyVac4 vaccine (Ag85B-TB10.4) as well as an *L. major* antigen, where adjuvanted protein also induced higher levels of IL-2 inducing memory/multifunctional T cells compared to the viral vector [46], [47]. It has been shown that prolonged exposure to antigen is required for optimal T cell memory development [48], [49]. It could therefore be speculated that some viral vectors induce more effector-like cells because the vectors are cleared more rapidly than e.g. the CAF01 adjuvant, which is known to create an antigen depot at the site of injection [50] leading to a more efficient development of central memory CD4 T cells.

In terms of protection, we found that both the H28/H28 and H28/MVA28 vaccine strategies were highly protective against infection with M.tb. However, H28/H28 induced a significantly increased protection compared to H28/MVA28 in the mouse TB model indicating that the increased ability to produce IL-2 amongst vaccine specific T cell subsets generated in the H28/H28 group was protective. MVA28 alone induced the lowest protection in the mouse model, demonstrating that MVA expressing H28 is best suited as a booster vaccine (fig. 3A and S4).

Thus, the H28/H28 mice showed the highest level of protection, which in turn correlated with the presence of certain T cell subsets pre and/or post infection. In terms of pre infection responses the TNF-α^+^IL-2^+^ central memory-like CD4 subset showed the highest correlation with the level of bacteria in the lungs 6 weeks after infection (fig. 4A). After the infection more subsets correlated with protection (fig. 4B). Thus, both TNF-α^+^IL-2^+^, IFN-γ^+^TNF-α^+^IL-2^+^ and IFN-γ^+^TNF-α^+^ CD4 T cells showed significant correlation, and it could be speculated that all T cell subsets arise from one pre infection subset, the TNF-α^+^IL-2^+^ subset, which is able to both provide post infection effector T cell subsets as well as maintaining a central memory pool of T cells. In agreement with these data we recently showed that boosting BCG with H4 (Ag85B-TB10.4) increased protection against infection with M.tb. which in turn correlated with increased percentage of IFN-γ^+^TNF-α^+^IL-2^+^ and TNF-α^+^IL-2^+^ CD4 T cells [40]. The importance of IFN-γ^+^TNF-α^+^IL-2^+^ T cells is not surprising considering the well documented role of IFN-γ in controlling growth of M. tuberculosis in macrophages [9], [12] and the high per-cell cytokine production as well as proliferative and self-sustaining capacity of this T cell subset [18]. In addition, the TNF-α^+^IL-2^+^ cells have been proposed to constitute a central memory phenotype [18], and these cell subsets may be of particular importance during a chronic infection since the central memory T cell pool is one of the subset of T cells that is exhausted during a persistent M.tb infection. Thus, TNF-α^+^IL-2^+^ T cells may function as a reservoir of new and functional effector T cells that can replace terminally differentiated and exhausted effector T cells during chronic infections [51], and it could be speculated that immune cell homeostasis is required for optimal protection. Furthermore, our data suggests that a low ability of the MVA vector to induce TNF-α/IL-2 expressing central memory-like CD4 T cells could be related to the failure of the MVA85A-vaccine in a recent phase 2b clinical trial [29].

Concerning CD8 T cells, MVA28 itself was not found to be an efficient inducer of CD8 T cells, and the apparent requirement of priming with an adjuvanted vaccine for a strong viral CD8 T cell induction has been observed previously with adenovirus expressed Ag85B-TB10.4 [46]. However, even though the H28/MVA28 prime boost strategy was the best strategy for inducing a CD8 T cell response, this did not translate into improved protection compared to CAF01 adjuvanted H28 alone. In support of this lack of correlation there are several examples that CD8 T cells in the mouse model does not seem to play a major protective role [9], [10], [40]. However, this does not rule out an important function of these cells in humans or in the NHP model. CD4 or CD8 T cells were not studied in the NHP model presented here (due to technical limitations).

The macaque TB model recapitulates the spectrum of pathology seen in human tuberculosis and the monkeys can develop the full spectrum of disease seen in humans [44], [52]. Therefore several vaccine efficacy studies have been published using the cynomolgus macaque model of infection [53], [54], [55], [56]. We also tested our vaccine strategies in the macaque TB animal model and decided to let the experimental setup reflect how we envision the most realistic human clinical trial. We therefore tested the vaccine strategies as BCG booster vaccines and used the adjuvant IC31®, which is presently in clinical trials with other TB vaccines [32]. Our data showed that in the macaque model both the BCG-H28/H28 and BCG-H28/MVA28 regimens were more protective than BCG alone, as demonstrated by the containment of infection, efficient prevention of clinical disease, improved overall survival of the animals and reduced pathological changes at necropsy (fig. 5). The efficient containment of subclinical infection in the H28 groups resulted in a limited disease spread and several animals had minimal disease and pathology at necropsy (fig. 5). This finding is different from other vaccines that have been tested in non-human primates where substantial pathology has been observed also in BCG-boosted groups [55], in some cases even at a higher level than found in animals only receiving BCG [31]. Even though both boosted groups (BCG-H28/H28 and BCG-H28/MVA28) were effectively protected against infection with M.tb, important differences were nevertheless observed between the groups. Thus, BCG-H28/H28 vaccinated animals showed the lowest pathology score in the lungs, the lowest dissemination to other organs and the highest number of symptom-free animals (X-ray) in the very late stages of infection.

Taken together, by boosting BCG we were able to delay and reduce clinical disease and pathology and increase survival. This was clearly seen in both boosted groups, although to a higher degree (though not statistically significant) in the group boosted twice with H28 in IC31®.

### Concluding Remarks and Implications

Although we did not perform identical analysis in the two models, it is worth noting that when the read-outs could be compared (i.e. in terms of protection) the different vaccine strategies gave similar results in both models. In the mouse model we intentionally chose priming with the experimental vaccines over a BCG boost so that we did not risk that a BCG-prime would mask the endogenous potential and T cell inducing profile of the experimental vaccines. Thus, both vaccine strategies protected in mouse and monkeys, but the H28/H28 strategy seemed slightly more efficient in both models, and in the mouse model TNF-α/IL-2 expressing T cells seemed particularly important for protection. In the NHP model, T cell analysis was not performed, and future studies will have to establish the correlation between T cell phenotypes and protection in this model. We are presently performing these experiments in a collaborative project in monkeys vaccinated with subunit vaccines.

It has been suggested that the mouse model of tuberculosis does not accurately reflect the complete picture of how protective immune responses against tuberculosis develop in humans, and that better models of this disease are needed. In the case of CD8 T cells this certainly seem to be the case as CD8 T cells seem to play a more robust role in the NHPs [1] which, in contrast to mice, also express the CD8 T cell effector molecule granulysin [2]. Moreover, the pathology in *M. tuberculosis*-infected macaques resemble human tuberculosis more closely than the pathology observed in M.tb infected mice [57]. Thus, in contrast to Non-human primates, granulomas in most mouse strains comprise loose non-necrotic aggregates, instead of the necrotic granulomas that are a hallmark of human tuberculosis. However, as we observed a good correlation between the two models, this indicated that although the mouse model is not the optimal model in terms of understanding tubercolusis pathology [9], [10], [40], it is a good model for evaluating TB vaccine efficacy against an acute infection. In contrast, the NHP model should be expected to be a better model for testing vaccines against TB reactivation, as mice do not develop a latent infection [57]. Based on our findings in the mouse model we suggest that a protective vaccine against infection with M.tb should primarily possess the ability to induce (and maintain) CD4 phenotypes expressing TNF-α/IL-2. Finally, in terms of the vaccine antigen, H28 shows great promise as a new efficient vaccine against TB to be tested in clinical trials.

## Supporting Information

Figure S1 **CD4 TB10.4 responses and Ag85B CD8 responses post vaccination.** One week after the second and final immunization splenocytes were analyzed for intracellular cytokine production by CD4 (A) or CD8 (B) T cells following stimulation with TB10.4 (A) or Ag85B (B). Mice were immunized as described in figure 2. The samples analyzed are the same as in figure 2. Bars represent percentages of CD4 or CD8 T cells from individual spleens from 4 mice per group producing any combination of IFN-γ, TNF-α or IL-2 as indicated below the graphs. Background levels obtained in media-stimulated samples have been deducted. Pie charts represent the relative distribution within the antigen specific CD4 or CD8 T cells pools of subsets producing different cytokine combinations as shown in the histograms.
(TIF)

Figure S2 **The expression of IFN-γ and IL-2 per single antigen specific CD4 T cell.** A, the level of IFNγ (left panels) or IL-2 (right panels) produced per CD4 T cell after Ag85B (A) or TB10.4. B, stimulation was measured by mean fluorescence intensity (MFI) of fluorescent staining by the relevant anti-cytokine antibody. Bars represent mean MFI of cytokines produced by antigen specific splenocytes from the same 4 individual mice and experiment as shown in figure 2. Bars represent means and SEM of four individual mice per group. MFI analysis was performed on cytokine-positive (IFN-γ or IL-2) CD4 T cells.
(TIF)

Figure S3 **CD4 TB10.4 responses and Ag85B CD8 responses in lungs 6 weeks post infection.** Mice were immunized with recombinant vaccines as described in figure 2. Six weeks after the final vaccinations mice were subjected to aerosol infection with M.tb, and intracellular cytokine analysis was performed by flow cytometry on lung cells six weeks after infection. The samples analyzed are the same as in figure 3. Cells were stimulated with TB10.4 for CD4 response analysis (A) and Ag85B for CD8 response analysis (B). Bars represent percentages of CD4 (A) or CD8 T cells (B) from individual lungs from 3 mice per group producing any combination of IFN-γ, TNF-α or IL-2 as indicated below the graphs. Background levels obtained in media-stimulated samples have been deducted. Pie charts represent the relative distribution of CD4 or CD8 T cells subsets producing different cytokine combinations as shown in the histograms.
(TIF)

Figure S4 **Meta analysis of four independent experiments.** Mice were immunized in two weeks intervals with recombinant vaccines. Six weeks after the final vaccination mice were subjected to aerosol infection with M.tb., and the bacterial burden was determined 6 weeks after infection. Bars represent mean and SEM of estimated log_10_ protection values from N = 22–23 mice per group from a total of four individual experiments. Log_10_ protection values were obtained by deducting log_10_ CFU values for each individual mouse from the mean of the none-vaccinated group in each separate experiment. *p<0.05, **p<0.01, ***p<0.001 as indicated in the graph using one-way ANOVA and Newman-Keul’s post test for multiple comparisons.
(TIF)

Figure S5 **Correlation between CFU and pre- and post infection CD8 responses.** A, the splenic total IFN-γ CD8 T cell response obtained using ICS and flow cytometry one week after immunizations shown in figure 2 (TB10.4-stimulation) was correlated to the corresponding mean log_10_ CFU value obtained in the lungs six weeks after infection and shown in figure 3 within each group. Points represent mean percentage and SEM (vertical) of CD8 T cells producing IFN-γ in response to stimulation with indicated antigens from 4 spleens per group plotted on the y-axis and mean and SEM (horizontal) log_10_ CFU values of individual mice on the x-axis. Each point represents one group as indicated in the graph. B, the pulmonary total IFN-γ CD8 T cell response obtained using ICS and flow cytometry six weeks after infection shown in figure 3 (TB10.4-stimulation) was correlated to the corresponding mean log_10_ CFU value shown in figure 3A within each group. Points represent individual mean and SEM values as described in A. *p<0.05, **p<0.01, using Pearson’s product-moment correlation coefficient (r) and correlation test.
(TIF)

Table S1 **Clinical parameters for individual animals after challenge.** Non-vaccinated, BCG vaccinated and BCG vaccinated H28 or H28/MVA28 boosted monkeys were infected with M.tb (500 CFU) and evaluated from 0 to 64 weeks (every four weeks) post challenge. Weight and temperature of the animals were determined at the indicated time points. Erythrocyte sedimentation rates were determined on whole blood. X-ray: the animals were scored according the following definitions: (−): negative, SN: scattered nodules, BP(u): unilateral bronchopneumonia, BP(b): bilateral bronchopneumonia, BP/Con: Bronchopneumonia with consolidation of lung, BP/At: Bronchopneumonia with Atelectasis.
(XLSX)

Table S2 **Pathology scores at necropsy after challenge.** Gross pathology in lungs and other organs. Pathology scores (maximum score is 10) given as individual organ scores of lungs and other organs in the vaccine and control groups after infection with *M. tuberculosis*.
(XLSX)
