# Supplementary figures and images for: Comparing Adjuvanted H28 and Modified Vaccinia Virus Ankara Expressing H28 in a Mouse and a Non-Human Primate Tuberculosis Model
Source: PLoS One. 2013 Aug 19;8(8):e72185. doi: 10.1371/journal.pone.0072185 (PMC3747044; doi:10.1371/journal.pone.0072185)

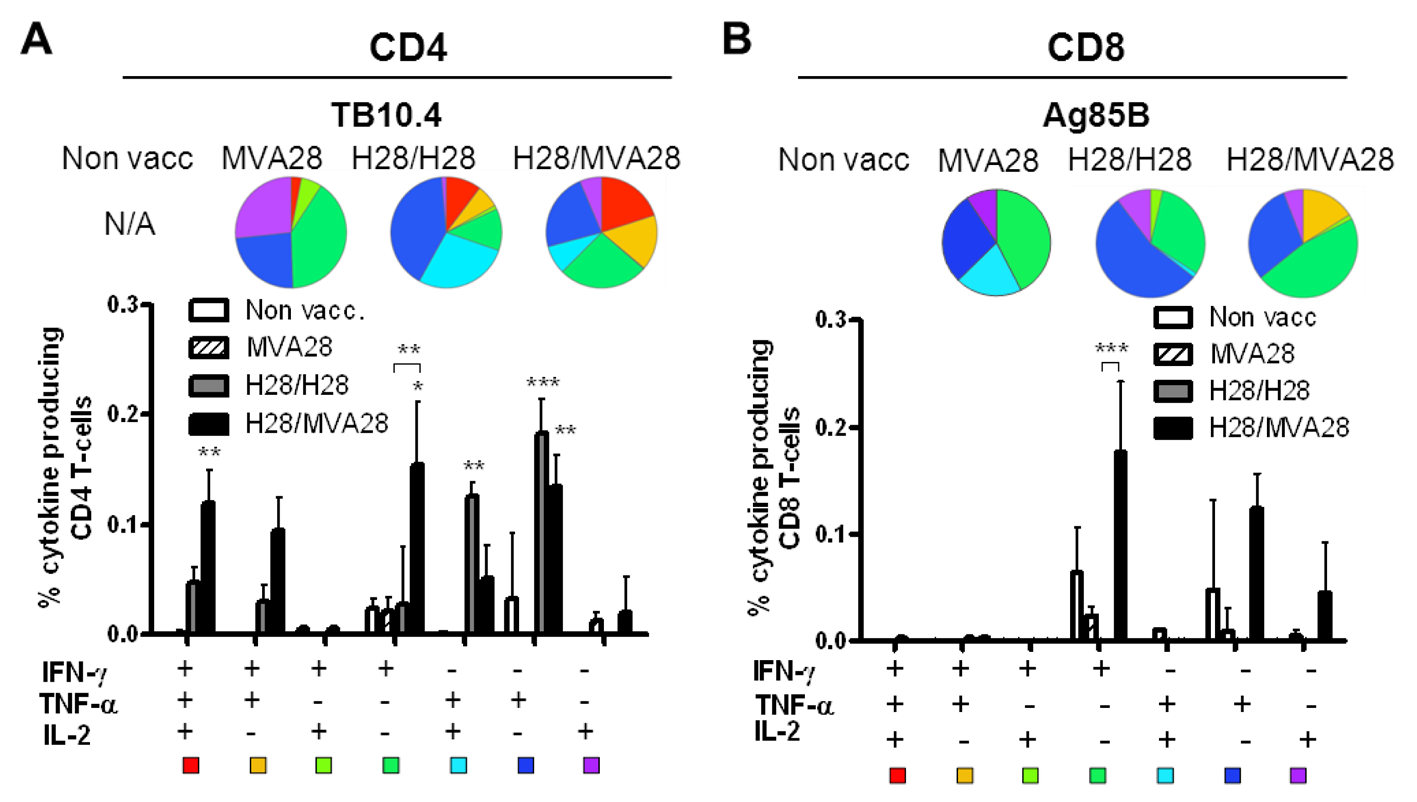

Supplement: Figure S1 — CD4 TB10.4 responses and Ag85B CD8 responses post vaccination. One week after the second and final immunization splenocytes were analyzed for intracellular cytokine production by CD4 (A) or CD8 (B) T cells following stimulation with TB10.4 (A) or Ag85B (B). Mice were immunized as described in figure 2. The samples analyzed are the same as in figure 2. Bars represent percentages of CD4 or CD8 T cells from individual spleens from 4 mice per group producing any combination of IFN-γ, TNF-α or IL-2 as indicated below the graphs. Background levels obtained in media-stimulated samples have been deducted. Pie charts represent the relative distribution within the antigen specific CD4 or CD8 T cells pools of subsets producing different cytokine combinations as shown in the histograms. (TIF) [file pone.0072185.s001.tif]

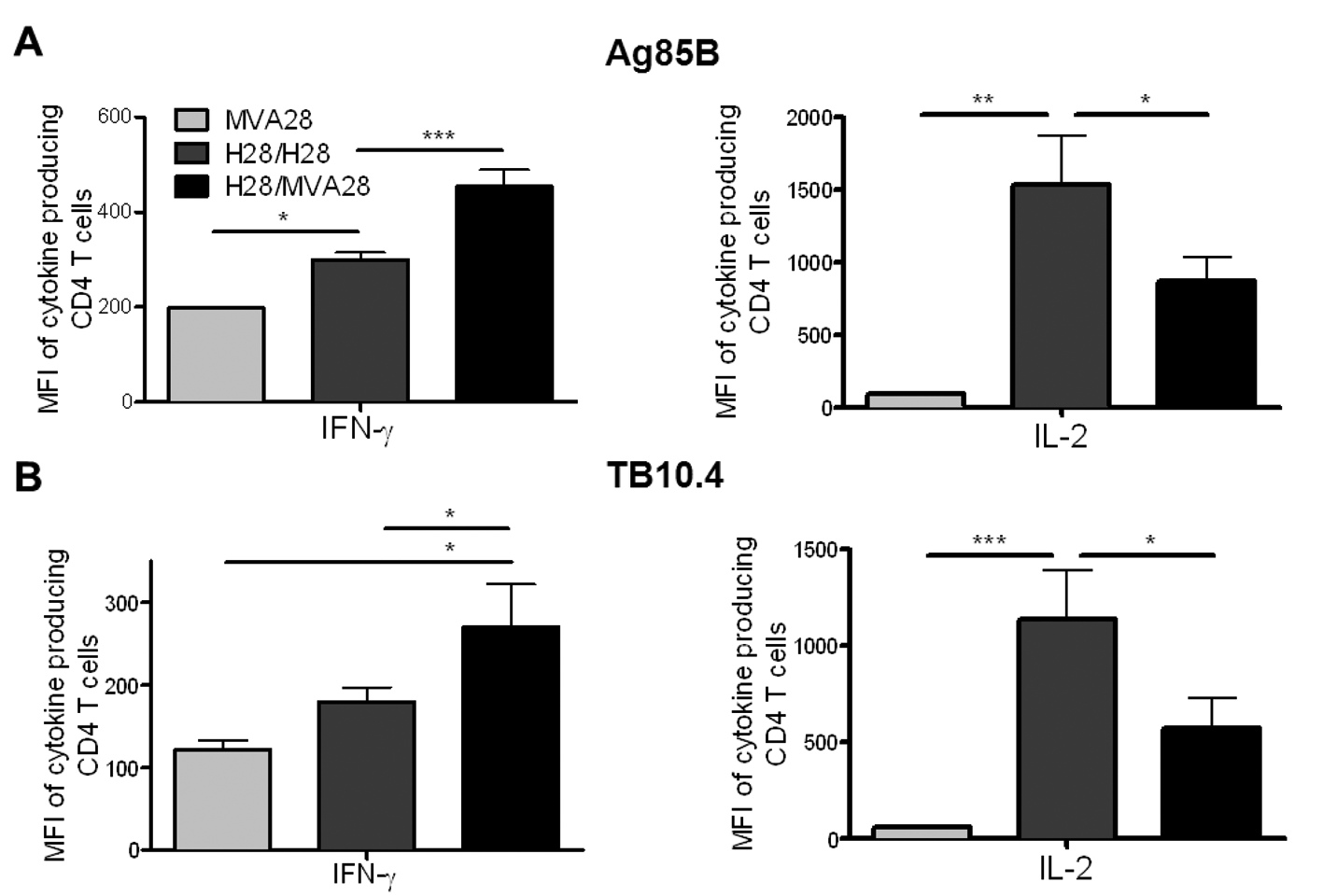

Supplement: Figure S2 — The expression of IFN-γ and IL-2 per single antigen specific CD4 T cell. A, the level of IFNγ (left panels) or IL-2 (right panels) produced per CD4 T cell after Ag85B (A) or TB10.4. B, stimulation was measured by mean fluorescence intensity (MFI) of fluorescent staining by the relevant anti-cytokine antibody. Bars represent mean MFI of cytokines produced by antigen specific splenocytes from the same 4 individual mice and experiment as shown in figure 2. Bars represent means and SEM of four individual mice per group. MFI analysis was performed on cytokine-positive (IFN-γ or IL-2) CD4 T cells. (TIF) [file pone.0072185.s002.tif]

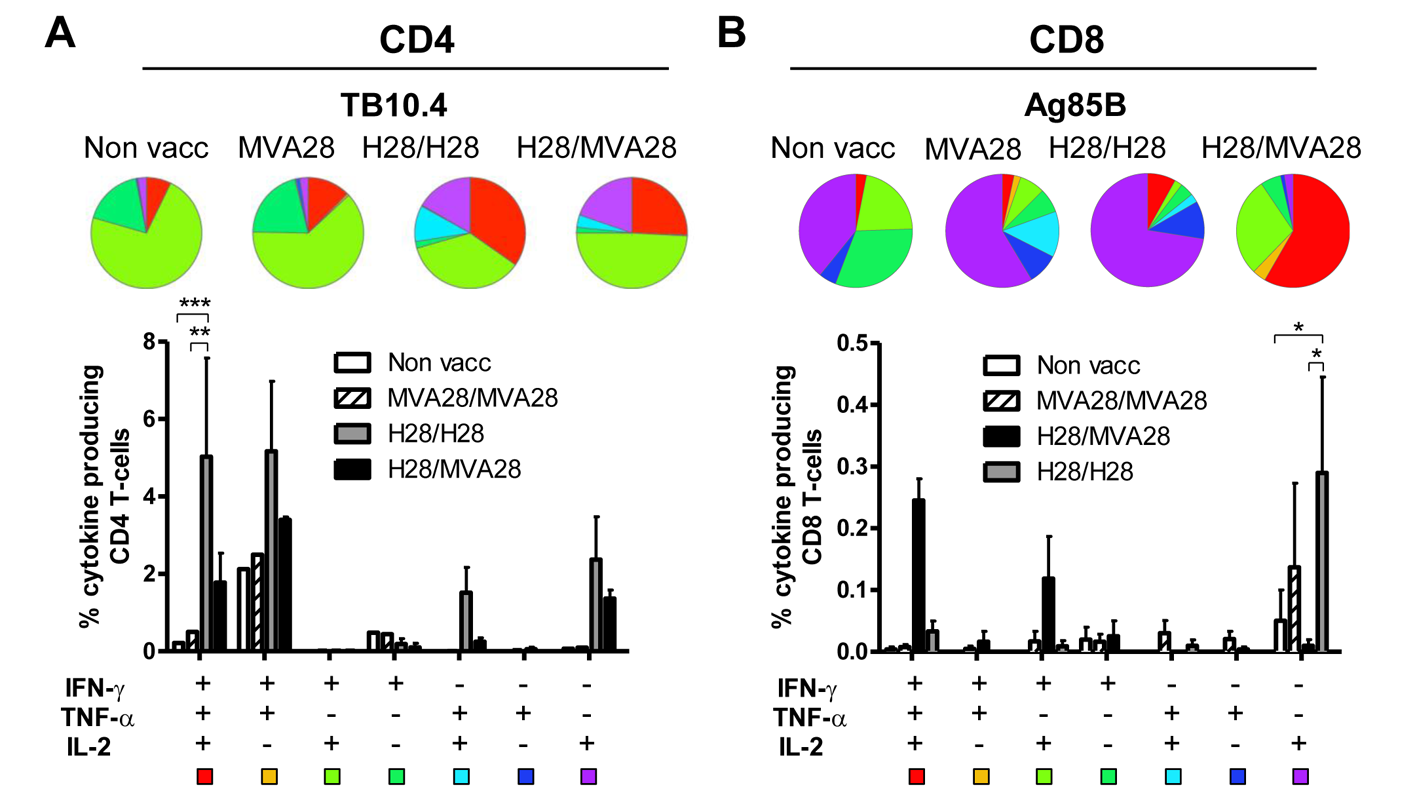

Supplement: Figure S3 — CD4 TB10.4 responses and Ag85B CD8 responses in lungs 6 weeks post infection. Mice were immunized with recombinant vaccines as described in figure 2. Six weeks after the final vaccinations mice were subjected to aerosol infection with M.tb, and intracellular cytokine analysis was performed by flow cytometry on lung cells six weeks after infection. The samples analyzed are the same as in figure 3. Cells were stimulated with TB10.4 for CD4 response analysis (A) and Ag85B for CD8 response analysis (B). Bars represent percentages of CD4 (A) or CD8 T cells (B) from individual lungs from 3 mice per group producing any combination of IFN-γ, TNF-α or IL-2 as indicated below the graphs. Background levels obtained in media-stimulated samples have been deducted. Pie charts represent the relative distribution of CD4 or CD8 T cells subsets producing different cytokine combinations as shown in the histograms. (TIF) [file pone.0072185.s003.tif]

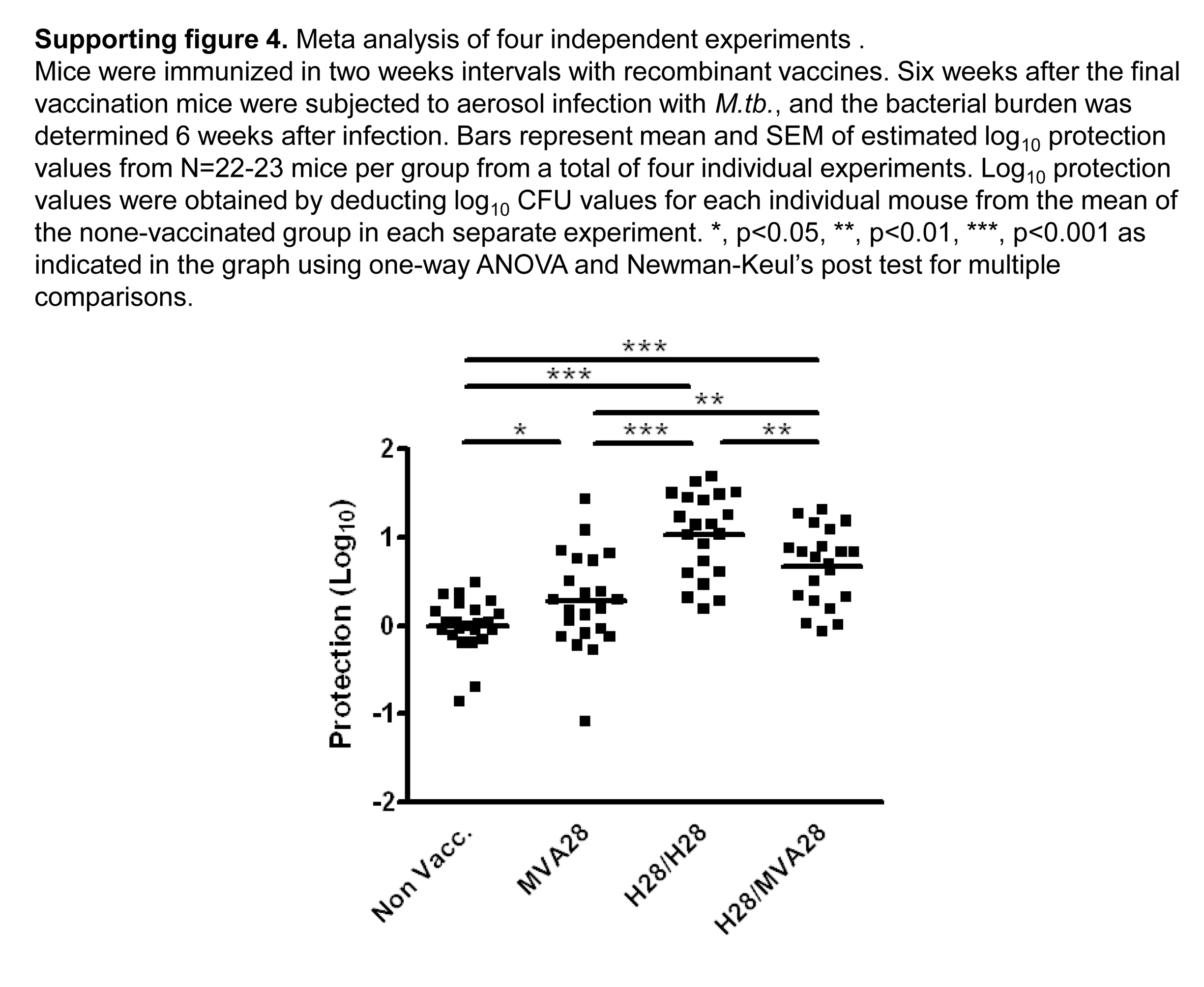

Supplement: Figure S4 — Meta analysis of four independent experiments. Mice were immunized in two weeks intervals with recombinant vaccines. Six weeks after the final vaccination mice were subjected to aerosol infection with M.tb., and the bacterial burden was determined 6 weeks after infection. Bars represent mean and SEM of estimated log10 protection values from N = 22–23 mice per group from a total of four individual experiments. Log10 protection values were obtained by deducting log10 CFU values for each individual mouse from the mean of the none-vaccinated group in each separate experiment. *p<0.05, **p<0.01, ***p<0.001 as indicated in the graph using one-way ANOVA and Newman-Keul’s post test for multiple comparisons. (TIF) [file pone.0072185.s004.tif]

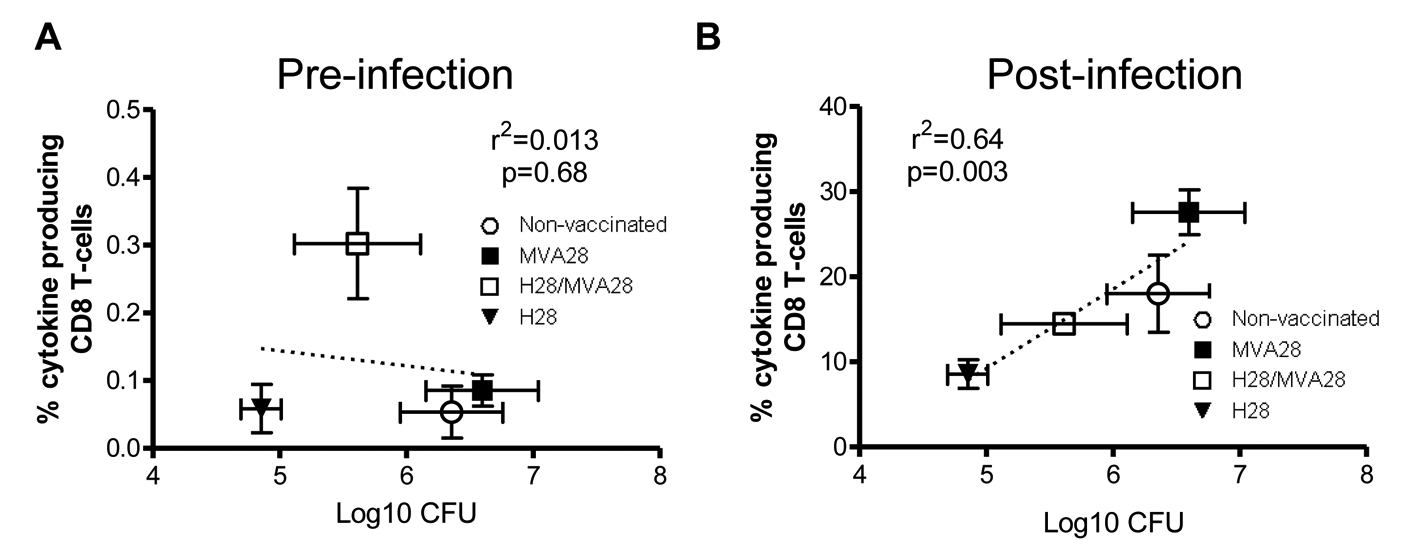

Supplement: Figure S5 — Correlation between CFU and pre- and post infection CD8 responses. A, the splenic total IFN-γ CD8 T cell response obtained using ICS and flow cytometry one week after immunizations shown in figure 2 (TB10.4-stimulation) was correlated to the corresponding mean log10 CFU value obtained in the lungs six weeks after infection and shown in figure 3 within each group. Points represent mean percentage and SEM (vertical) of CD8 T cells producing IFN-γ in response to stimulation with indicated antigens from 4 spleens per group plotted on the y-axis and mean and SEM (horizontal) log10 CFU values of individual mice on the x-axis. Each point represents one group as indicated in the graph. B, the pulmonary total IFN-γ CD8 T cell response obtained using ICS and flow cytometry six weeks after infection shown in figure 3 (TB10.4-stimulation) was correlated to the corresponding mean log10 CFU value shown in figure 3A within each group. Points represent individual mean and SEM values as described in A. *p<0.05, **p<0.01, using Pearson’s product-moment correlation coefficient (r) and correlation test. (TIF) [file pone.0072185.s005.tif]
